# Low-Density Lipoprotein Contributes to Endometrial Carcinoma Cell Proliferation, Migration, and Invasion by Activating the JAK–STAT Signaling Pathway

**DOI:** 10.1155/2023/4015167

**Published:** 2023-10-20

**Authors:** Lifan Shen, Chen Zhang, Kaiying Cui, Xin Liang, Genhai Zhu

**Affiliations:** ^1^Department of Gynecology, Hainan General Hospital (Hainan Affiliated Hospital of Hainan Medical University), 19th Xiuhua Road, Xiuying, Haikou 570000, China; ^2^Department of Central Lab, Hainan General Hospital (Hainan Affiliated Hospital of Hainan Medical University), Haikou, China

## Abstract

**Background:**

Cholesterol-rich low-density lipoprotein (LDL) particles have been demonstrated to regulate breast cancer cell proliferation and migration, but their biological function and relevant mechanisms in endometrial carcinoma (EC) remain unclear

**Methods:**

Serum and tissue samples were collected from EC patients (*n* = 50) and patients with benign endometrial hyperplasia (*n* = 50). Ishikawa and RL95-2 cells were stimulated with different concentrations of LDL, followed by treatment with a JAK2 inhibitor (SD-1029). LDL concentrations were determined by ELISA. The *in vitro* biological behavior of cells was examined using the CCK-8 assay, EdU staining, and Transwell assay. The tumorigenicity of LDL *in vivo* was examined using a xenograft mouse model. western blotting, immunofluorescence, and immunohistochemistry studies were performed to measure related protein expression.

**Results:**

The LDL concentrations and levels of p-JAK2 and p-STAT3 expression were elevated in the clinical samples. Similar trends in expression were detected in EC cells after LDL stimulation. LDL treatment significantly promoted EC cell proliferation, migration, and invasion, and also upregulated p-JAK2 and p-STAT3 expression in a dose-dependent manner. Moreover, SD-1029 dramatically blocked the LDL-mediated effects on EC cells. Intravenous injection of LDLs promoted tumor growth in the xenograft nude mice, and also increased p-JAK2, p-STAT3, and Ki-67 expression, and downregulated caspase-3 expression.

**Conclusions:**

These findings indicate that LDLs exert an oncogenic effect in EC cells by activating the JAK/STAT signaling pathway, and also suggest the JAK/STAT pathway as a possible therapeutic target for EC.

## 1. Introduction

Endometrial carcinoma (EC) originates from the uterus epithelium and is one of the most prevalent gynecologic malignant tumors with high rates of morbidity and mortality [1, 2]. Despite advances made in standard therapeutic approaches, the disease recurrence and death rates of patients with advanced stage EC remain relatively high [3, 4]. Hence, it is important to investigate the molecular mechanisms of EC progression and identify signaling pathways that can be used for its diagnosis and treatment.

It is well known that a metabolic disorder can increase the risk for tumorigenesis. This is because tumors have higher energy demands and rates of biosynthesis that are needed to support uncontrolled cancer cell growth and metastasis [5]. Oxysterols, steroid hormones, and cholesterol are essential for cell growth under conditions of abnormal metabolism [6]. Cholesterol-rich lipoprotein particles in peripheral blood serve to transfer cholesterol from the liver to peripheral tissues and organs [7, 8]. Emerging evidence indicates the involvement of LDLs, oxidized low-density lipoproteins (ox-LDLs), and LDL receptors in the development of cancer [9]. Deng et al. [10] showed that preoperative LDL serum levels play an important role in predicting the prognosis of esophageal squamous cell carcinoma (ESCC), due to their promotive effects on lymphatic metastasis. Elevated serum ox-LDL levels may be correlated with an advanced stage of prostate cancer and its lymph node metastasis [11]. In addition, LDL levels have been shown to be correlated with small-cell lung cancer prognosis [12] and breast cancer disease-free survival time [13]. Moreover, preclinical data have indicated that LDLs play an important role in the increased growth and proliferation of various cancers by modulating different signaling pathways [14–16]. Among those pathways, the Janus kinase/signal transducer and activator of transcription JAK–STAT signaling pathway, consisting of the JAK family (JAK1-3 and TYK2) and STAT family (STAT1-4, STAT5a, STAT5b, and STAT6) has been of great interest when studying human malignancies [17, 18]. Lu et al. [19] reported that LDLs affect breast cancer cell growth and transformation via the JAK–STAT signaling pathway by regulating STAT3. Therefore, we hypothesized that LDLs may play an important role in EC tumorigenesis and their regulatory effect may be correlated with the JAK–STAT signaling pathway.

To test our hypothesis, we first determined the concentrations of LDL and levels of JAK–STAT signaling-related proteins in the clinical samples. Next, we investigated the effects of LDLs on EC cellular behaviors (proliferation, migration, and invasion) and tumor growth *in vivo*. Furthermore, we explored whether the JAK–STAT signaling pathway was involved in LDL-mediated EC cell functions.

## 2. Materials and Methods

### 2.1. Clinical Samples

Samples of EC tissue (*n* = 50) and benign endometrial hyperplasia tissue (*n* = 50) were collected from patients at the Hainan General Hospital between 2020 and 2022. Each patient provided their signed written informed consent for study participation. In addition, corresponding blood samples were obtained from the patients prior to receiving any treatment. The blood samples were initially centrifuged for 15 min (3,000 *g*) at 4°C, and subsequently centrifuged for another 15 min (10,000 *g*) at 4°C to obtain serum samples. The study protocol was approved by the Ethics Committee of Hainan General Hospital (No. 2022-594).

### 2.2. Cell Culture and Treatment

Two EC cell lines (Ishikawa and RL95-2) were provided by the American Type Culture Collection (ATCC; Manassas, VA, USA). The cells were cultured in DMEM/F12 medium (Gibco, Waltham, MA, USA) containing 10% FBS (Gibco) at 37°C in an incubator with 5% CO_2_ atmosphere. The EC cells were stimulated by treatment with different concentrations of LDL (0.5, 1.5, and 3.0 *μ*g/mL) for 24 hr. To investigate the function of the JAK–STAT signaling pathway, the EC cells were first treated with SD-1029 (a JAK2 inhibitor; Sigma-Aldrich, St Louis, MO, USA), followed by 24 hr of stimulation with 3.0 *μ*g/mL LDL.

### 2.3. Enzyme-Linked Immunosorbent Assay (ELISA)

The concentrations of LDL in serum samples and the culture media of EC cells were measured using ELISA kits (R&D Systems, Minneapolis, MN, USA). Each assay was performed in triplicate.

### 2.4. Immunofluorescence

EC cells from different groups were inoculated into 12-well plates and then fixed with 4% paraformaldehyde for 15 min; after which, they were washed with PBS and blocked with 10% sheep serum for 1 hr at 37°C. Next, the cells were permeabilized with 0.5% Triton X-100 and incubated overnight at 4^o^C with primary antibodies against p-JAK2 and p-STAT3. After being washed three times with PBS, the cells were incubated for 2 hr with AlexaFluor594 conjugated secondary antibody in the dark at room temperature. Finally, the cellular nuclei were stained for 10 min with DAPI (Sigma-Aldrich) at room temperature, and the cells were observed with an inverted fluorescence microscope.

### 2.5. Cell Counting Kit-8 (CCK-8) Assay

Cell viability was determined using the CCK-8 (Dojindo, Kumamoto, Japan). Each assay was conducted in triplicate at 37°C, and using 10 *µ*L of CCK-9 solution. The absorbance of each assay well at 450 nm was detected with a microplate reader.

### 2.6. EdU Staining Assay

Cells from different groups were cultured in 96-well plates for 24 hr at 37°C. The following day, the cells were incubated for 2 hr with 50 *µ*L of 5-ethynyl-2′-deoxyuridine (EdU, Beyotime, Shanghai) solution at 37°C, and then fixed for 15 min with 4% paraformaldehyde at room temperature. Next, the cellular nuclei were stained with DAPI solution (Sigma-Aldrich), and images of EdU-positive cells were acquired under a microscope.

### 2.7. Transwell Assay

EC cell migration and invasion capabilities were evaluated using transwell assay plates (#3422, Corning, Corning, NY, USA). In brief, cells from different groups were suspended in serum-free media and transferred into the upper chambers of transwell plates for migration assays or inoculated into Matrigel (#356234, Corning, USA) coated transwell chambers for invasion assays. The lower chambers were filled with 600 *µ*L of medium containing 20% fetal bovine serum as a chemoattractant. After 24 hr of incubation at 37°C, the migrated and invasive cells in the lower chambers were fixed with 4% paraformaldehyde for 15 min, stained with 0.1% crystal violet for 30 min, and subsequently photographed and counted under a microscope.

### 2.8. Tumor Xenograft Assay

After gaining approval from the Ethics Review Committee of the Department of Laboratory Animal Science of China Medical University, animal experiments were performed to investigate the oncogenic role of LDL *in vivo*. Male C57BL/6 mice (age range, 4–6 weeks; *n* = 20) were purchased from Vital River Laboratories (Beijing, China) and housed in a specific pathogen-free environment. Next, 2 × 10^7^ Ishikawa or RL95-2 cells in 100 mL of dimethyl sulfoxide (DMSO) were subcutaneously injected into the subcutaneous tissues of the nude mice to establish xenograft tumor mouse models. After inoculation of the tumor cells, LDLs were intravenously injected into the mice every 3 days. Next, the mice were divided into the following four groups, with five mice in each group: Ishikawa control, Ishikawa LDL, RL95-2 control, and RL95-2 LDL. Control mice were treated with an equivalent amount of DMSO. When a tumor could be visually observed, its volume was calculated every 7 days using the following formula: *L* × (*W*)^2^/2, where *L* is tumor length and *W* is tumor width. When the tumor's diameter reached 2 cm, the mouse was sacrificed and the tumor tissue was excised, measured, and further analyzed.

### 2.9. Western Blot Analysis

Total proteins were extracted with RIPA lysis buffer (Sigma-Aldrich), and the protein concentration in each extract was determined using a BCA Quantification Kit (Beyotime). Next, an equal amount of protein from each extract was separated by 12% SDS-PAGE, and the protein bands were transferred onto PVDF membranes, which were subsequently blocked with 5% fat-free milk at room temperature for 2 hr. Subsequently, the protein bands were probed with primary antibodies (Abcam, Cambridge, UK) against JAK2, p-JAK2, STAT3, p-STAT3, and *β*-actin at 4°C overnight; after which, the membranes were incubated with HRP-conjugated goat anti-rabbit secondary antibodies for 2 hr at room temperature. The immunostained protein bands were detected with an enhanced Chemiluminescence Kit (Millipore, Burlington, MA, USA).

### 2.10. Immunohistochemistry

Samples of mouse tumor tissue were immediately fixed in 4% paraformaldehyde for 24 hr, embedded in paraffin, and cut into 5 *μ*m sections. The sections were then deparaffinized with xylene, rehydrated with ethanol, and repaired with citric acid. After blocking endogenous peroxidase activity, the tissue sections were incubated overnight at 4°C with primary antibodies against caspase-3 (#9662, Cell Signaling Technology, Danvers, MA, USA) and Ki-67 (#9129, Cell Signaling Technology), and subsequently washed with PBS. The sections were then incubated with a goat anti-rabbit secondary antibody for 30 min at 37°C, counterstained with hematoxylin, and examined under a light microscope (Olympus, Tokyo, Japan).

### 2.11. Statistical Analysis

All statistical data were analyzed using GraphPad Prism 8 software (GraphPad Prism, Inc., La Jolla, CA, USA), and quantitative data were presented as a mean value ± standard deviation (SD). Comparisons between two groups were performed using student's *t* test and comparisons between multiple groups were performed by one-way analysis of variance (ANOVA), followed by Dunnett's or Turkey's post hoc test. A *p*-value <0.05 was considered to be statistically significant.

## 3. Results

### 3.1. Expression of LDL and the JAK/STAT Pathway in Samples from EC Patients

**Figure 1:**
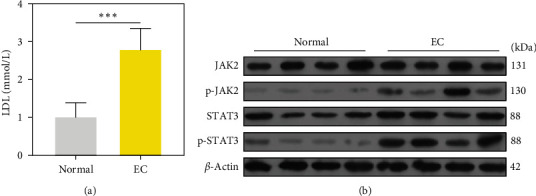
Expression of LDLs and the JAK/STAT pathway in samples from EC patients. (a) ELISA assays were performed to determine the concentrations of LDLs in serum samples from EC patients (*n* = 50) and benign endometrial hyperplasia patients (*n* = 50);  ^*∗∗∗*^*p* < 0.001; (b) The levels of JAK2, p-JAK2, STAT3, and p-STAT3 proteins were detected in tissue samples from EC patients (*n* = 50) and benign endometrial hyperplasia patients (*n* = 50).

To measure the levels of LDLs in EC, we collected serum samples from 50 EC patients and 50 patients with benign endometrial hyperplasia. ELISA results showed a significant increase in LDL concentrations in the serum samples from EC patients, when compared with samples obtained from benign endometrial hyperplasia patients (Figure 1(a)). In addition, we analyzed the levels of JAK/STAT pathway-related proteins by western blotting. As shown in Figure 1(b), the p-JAK2 and p-STAT3 protein levels were obviously elevated in the tissue specimens from EC patients, when compared to specimens form patients with benign endometrial hyperplasia. These results suggest that the abnormal levels of LDLs and JAK/STAT pathway proteins may be related to the genesis and development of EC ( ^*∗∗∗*^*p* < 0.001).

### 3.2. The Promotive Effects of LDL Treatment on EC Cell Proliferation, Migration, and Invasion

**Figure 2:**
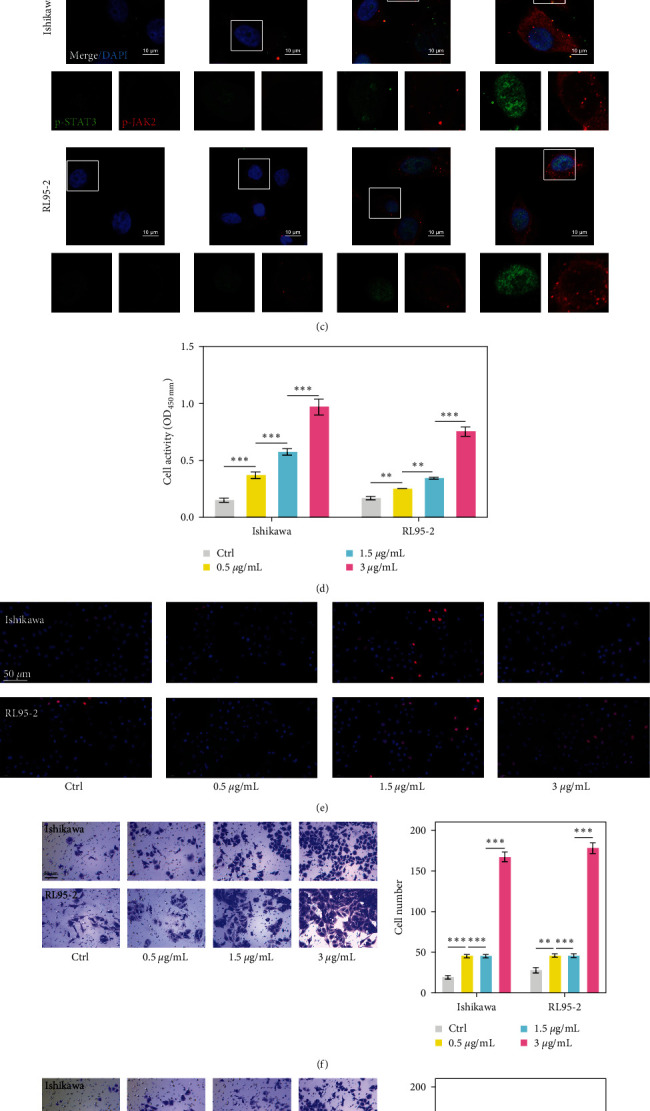
LDL treatment promoted EC cell proliferation, migration, and invasion in a dose-dependent manner. Ishikawa and RL95-2 cells were stimulated with 0.5, 1.5, and 3.0 *μ*g/mL of LDL for 24 hr. (a) LDL concentrations in the culture media of EC cells were determined by ELISA. (b) The levels of JAK2, p-JAK2, STAT3, and p-STAT3 proteins in EC cells were detected by western blotting. (c) p-JAK2 and p-STAT3 expression were determined by immunofluorescence staining. (d) CCK-8 assays and (e) EdU staining were performed to assess the viability and proliferative ability of Ishikawa and RL95-2 cells, respectively. (f, g) Transwell assays were used to evaluate the migration and invasion capabilities of Ishikawa and RL95-2 cells. Data are expressed as a mean value ± SD;  ^*∗*^*p* < 0.05,  ^*∗∗*^*p* < 0.01, and  ^*∗∗∗*^*p* < 0.001.

To investigate the biological function of LDLs in EC cells, Ishikawa and RL95-2 cells were treated with different concentrations of LDLs for 24 hr. ELISA results indicated that the LDL uptake by Ishikawa and RL95-2 cells became markedly elevated as the LDL concentration increased, suggesting a positive correlation between LDL uptake and LDL treatment concentration (Figure 2(a)). Meanwhile, LDL treatment gradually increased the p-JAK2 and p-STAT3 protein levels in Ishikawa and RL95-2 cells in a dose-dependent manner, as determined by western blotting (Figure 2(b)) and immunofluorescence staining (Figure 2(c)). Subsequently, a series of functional experiments were performed with Ishikawa and RL95-2 cells. CCK-8 assays showed that LDL treatment significantly increased the viability of Ishikawa and RL95-2 cells in a dose-dependent manner (Figure 2(d)). Moreover, EdU staining showed that Ishikawa and RL95-2 cell proliferation gradually increased as the LDL concentration increased (Figure 2(e)). Transwell assays revealed that LDL treatment significantly enhanced the migratory (Figure 2(f)) and invasion (Figure 2(g)) abilities of Ishikawa and RL95-2 in a dose-dependent manner. These results showed that LDL treatment promoted the proliferation, migration, and invasion of EC cells, and those effects were related to expression of the JAK/STAT pathway ( ^*∗*^*p* < 0.05,  ^*∗∗*^*p* < 0.01, and  ^*∗∗∗*^*p* < 0.001).

### 3.3. LDL Enhanced-EC Cell Proliferation, Migration, and Invasion by Regulating the JAK/STAT Signaling Pathway

**Figure 3:**
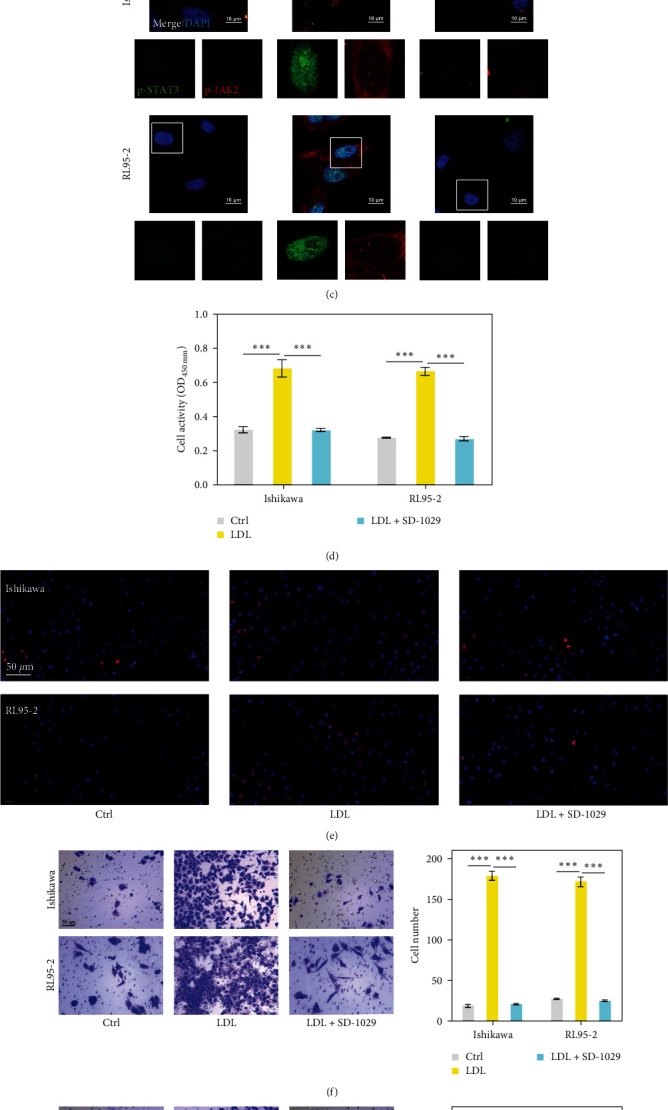
LDLs enhanced the proliferation, migration, and invasion of EC cells by regulating the JAK/STAT signaling pathway. Ishikawa and RL95-2 cells were treated with a JAK2 inhibitor (SD-1029), followed by 24 hr of stimulation with 3.0 *μ*g/mL LDL. (a) The concentrations of LDL in the culture media of EC cells were determined by ELISA. (b) The levels of JAK2, p-JAK2, STAT3, and p-STAT3 proteins in EC cells were detected by western blotting. (c) p-JAK2 and p-STAT3 expression were determined by immunofluorescence staining. (d) CCK-8 assays and (e) EdU staining were used to assess the viability and proliferative ability of Ishikawa and RL95-2 cells, respectively. (f, g) Transwell assays were performed to evaluate the migration and invasion abilities of Ishikawa and RL95-2 cells. Data are expressed as a mean value ± SD;  ^*∗∗*^*p* < 0.01 and  ^*∗∗∗*^*p* < 0.001.

To further clarify the role played by the JAK/STAT pathway in the process of LDL-mediated EC cell functions, Ishikawa and RL95-2 cells were treated with a JAK2 inhibitor (SD-1029), followed by 24 hr of stimulation with 3.0 *μ*g/mL LDL. As shown in Figure 3(a), when compared with the control groups, Ishikawa and RL95-2 cells treated with LDLs had significantly higher LDL concentrations, and treatment with SD-1029 did not affect those increases in LDL concentration. As expected, western blot assays (Figure 3(b)) and immunofluorescence staining (Figure 3(c)) showed that treatment with a JAK2 inhibitor (SD-1029) obviously reduced the levels of p-JAK2 and p-STAT3 expression in LDL-treated Ishikawa and RL95-2 cells, when compared with those levels in the LDL group. Functional assays showed that the LDL-induced increases in cell viability (Figure 3(d)), EdU-positive cells (Figure 3(e)), cell migration (Figure 3(f)), and cell invasion (Figure 3(g)) were significantly reversed after cotreatment with LDL and the JAK2 inhibitor (SD-1029) when compared to cells treated with LDL alone. These results further indicated that LDL treatment could promote the proliferation, migration, and invasion of EC cells by inducing expression of the JAK/STAT pathway ( ^*∗∗*^*p* < 0.01 and  ^*∗∗∗*^*p* < 0.001).

### 3.4. LDL-Promoted Tumorigenesis *In Vivo*

**Figure 4:**
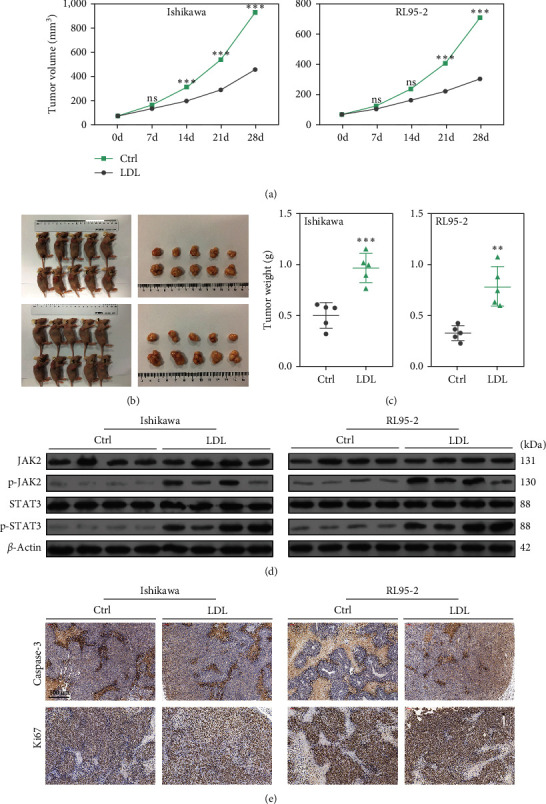
LDLs promoted tumorigenesis *in vivo*. Xenograft mouse models were established by injecting Ishikawa or RL95-2 cells into the subcutaneous tissues of nude mice, followed by intravenous injection of LDLs (*n* = 5 per group). (a) Changes in tumor volume were recorded on Days 7, 14, 21, and 28 after intravenous injection of LDLs;  ^*∗∗∗*^*p* < 0.001, ns: not significant. (b) The xenograft model mice (left panel) and representative images of the tumors formed in the mice (right panel). (c) Changes in tumor weight after intravenous injection of LDLs;  ^*∗∗*^*p* < 0.01 and  ^*∗∗∗*^*p* < 0.001. (d) Western blot analysis of JAK2, p-JAK2, STAT3, and p-STAT3 protein expression in the harvested mouse tumor tissues; *β*-actin served as control. (e) Representative immunohistochemistry images of caspase-3 and Ki-67 expression in mouse tumor tissues. Scale bar, 20 *μ*m.

Xenograft mouse models were established to verify the effects of LDLs on tumorigenicity *in vivo*. Ishikawa and RL95-2 cells were injected into the subcutaneous tissues of nude mice. Subsequent tumorigenicity assays revealed that intravenous injection of LDLs significantly increased the volumes of tumors created by the Ishikawa and RL95-2 cells over a period of 28 consecutive days, when compared with the tumors in control mice treated with an equal amount of DMSO (Figure 4(a)). When the tumors reached 2 cm in diameter, the mice were sacrificed and the tumor weights were recorded. As shown in Figures 4(b) and 4(c), both the size and weight of the tumors in mice intravenously injected with LDLs were obviously increased when compared with tumors in the respective control groups. Furthermore, we also analyzed the levels of p-JAK2 and p-STAT3 proteins in tissues of the mice. As shown in Figure 4(d), the levels of p-JAK2 and p-STAT3 proteins in tumor tissues from the LDL groups were significantly higher than those in tumor tissues from the DMSO groups. Immunohistochemistry assays showed that when compared with the control groups, caspase-3 expression was decreased and Ki-67 expression was increased in mice injected with the LDLs and Ishikawa or RL95-2 cells (Figure 4(e)). Thus, our *in vivo* experiments showed that LDLs could promote tumor formation in mice injected with EC cells, which was consistent with results from the *in vitro* studies ( ^*∗∗*^*p* < 0.01 and  ^*∗∗∗*^*p* < 0.001).

## 4. Discussion

With the increasing incidence of EC among younger obese women, targeted therapy has been shown to be a better treatment for EC than radiotherapy and/or chemotherapy with their inevitable side effects [20]. We found that LDL concentrations were significantly increased in serum samples from EC patients when compared with those concentrations in serum samples from benign endometrial hyperplasia patients. A high level of LDL receptor expression is closely related with a poor recurrence-free survival time in breast cancer patients [21]. Moreover, our studies with Ishikawa and RL95-2 EC cells showed that increases in LDL concentration resulted in corresponding dose-dependent increases in cellular LDL uptake, proliferation, migration, and invasion. Similarly, LDLs have been found to promote the proliferation and migration of certain breast cancer cells, including MDA-MB-468, 4T1, MCF-7, HS578T, and MDA-MB-231 cells [22]. Increased lipoprotein uptake and LDL receptor expression have been shown to promote tumor growth in preclinical pancreatic and breast cancer models [21, 23]. In fact, lipid metabolism is an important regulator of tumor growth, during which lipids can accumulate as a result of de novo synthesis or of transport from distant sources by circulating lipoproteins [24]. Elevated circulating cholesterol levels have been shown to promote cancer growth and progression in the clinical studies and preclinical models [14, 21, 23]. There is accumulating *in vivo* evidence that mice develop high-LDL levels when fed a high-cholesterol diet. Moreover, when mice fed a high-cholesterol diet are inoculated with breast cancer cells, those cells show an increased proliferative ability when compared to breast cancer cells inoculated into mice fed a normal diet [25–27]. In line with those studies, our results also showed that intravenous injection of LDLs remarkably promoted tumor growth in mice injected with EC cells. Hence, it is not difficult to understand that LDLs, which are cholesterol-rich lipoprotein particles, act as positive regulators in the promotion of EC progression.

Mechanistically, we not only observed activation of the JAK/STAT pathway in EC tissues, but also demonstrated that LDLs promoted EC cell proliferation, migration, and invasion, as well as tumor growth by upregulating the phosphorylation of JAK2 and STAT3. Actually, STAT3 activation correlates with the transcription of genes involved with cell proliferation and metastasis, as well as the biosynthesis of angiogenesis-related VEGF and matrix metalloproteinases (MMPs) [28]. A bioinformatics analysis showed that STAT family-associated genes can serve as prognostic markers and therapeutic targets for EC [29]. Additionally, proteins in the JAK/STAT pathway have been shown to be critical mediators of the leptin-stimulated proliferative response and the invasiveness of human EC cells [30]. Inhibition of the JAK/STAT pathway is involved in the anti-cancer effects of Tanshinone I on HEC-1-A cells [31]. With regard to the regulatory effect of LDLs on JAK/STAT signaling in human diseases, a microarray analysis indicated that ox-LDLs in atherosclerosis strongly affect the JAK-STAT signaling pathway by acting through STAT1 and STAT2 [32, 33]. In particular, Yang et al. [34] reported that ox-LDL induced JAK2 phosphorylation by enhancing the interaction between CD36 and JAK2 to further activate STAT3 signaling in bladder cancer. In breast cancer, LDLs were shown to affect cell growth and transformation induced by the JAK–STAT signaling pathway by regulating STAT3 [19]. When taken together, these findings suggest that LDLs promote EC progression by activating the JAK/STAT signaling pathway.

In summary, our data show that LDLs promote EC progression by regulating the JAK/STAT signaling pathway. These findings suggest the JAK/STAT signaling pathway as a promising therapeutic target for EC.

## Data Availability

All data are available from the corresponding author with reasonable request.
